# Sjogren’s Disease—Aspects of Clinical Disease Beyond Dry Eyes/Mouth

**DOI:** 10.3390/jcm15031189

**Published:** 2026-02-03

**Authors:** Simon J. Bowman

**Affiliations:** 1 The Sjogren’s Clinic, University Hospitals Birmingham NHS Foundation Trust, Birmingham B15 2TT, UK; simon.bowman@uhb.nhs.uk; 2 Department of Inflammation and Ageing, College of Medicine and Health, University of Birmingham, Birmingham B15 2TT, UK; 3 General NHS Rheumatology Practice, Milton Keynes University Hospital, Milton Keynes MK6 5LD, UK

**Keywords:** Sjogren’s, systemic features, review

## Abstract

Primary Sjogren’s Disease (SjD) is characterized by features of dryness arising from inflammation in the secretary glands, particularly the salivary and lachrymal glands. Generalized symptoms of fatigue and limb pain are very common but, in addition, about 40% of patients have one or more features of organ-specific systemic disease. This review goes through the background of SjD including diagnosis and classification, epidemiology, impact and investigations such as ultrasound and lip biopsy. It then focuses in detail on each of the systemic organ-specific features, principally using the European League against Rheumatism (EULAR) Sjogren’s Syndrome Disease Activity Index (ESSDAI) along with some non-ESSDAI domains, before concluding with comments on disease heterogeneity, treatment, vaccination, pregnancy and surgery along with observations on patient perspectives. The aim is to provide a general overview of these aspects of the disease to complement other chapters in this monograph.

## 1. Introduction

Sjogren(’s) Disease (SjD), previously ‘Sjogren’s syndrome’, is characterized by focal lymphocytic infiltration of the exocrine glands, leading in particular to dry eyes (keratoconjunctivitis sicca) and dry mouth (xerostomia) [1]. For patients with mild to moderate dryness symptoms who do not have extraglandular/systemic features or a severe form of associated fatigue, the condition may be manageable with modest lifestyle changes and simple interventions, but for those with moderate/severe symptoms, SjD can have a major adverse impact on quality of life [2]. Furthermore, for patients with systemic involvement, there is a significantly increased mortality risk in SjD (standardized mortality ratio = 1.7), with infections, malignancies (especially lymphoma), and cardiovascular disease as leading causes of death [3].

Although SjD is an autoimmune ‘primary’ rheumatic disease in its own right, the glandular features, with some disease-specific heterogeneity on histology, may be found ‘associated’ with other rheumatic disorders such as rheumatoid arthritis (RA), systemic lupus erythematosus (SLE) or systemic sclerosis (SSc) [4] (previously ‘secondary Sjogren’s syndrome’).

This review will set out the broad clinical parameters of the disease, particularly its extraglandular/systemic features, how SjD is diagnosed and how its varied features impact patient quality of life and clinical outcome.

## 2. Diagnosis and Classification

The diagnosis of primary SjD is principally based on classification criteria, in particular the American College of Rheumatology (ACR)/European League against Rheumatism (EULAR) Classification Criteria (AECC) published in 2016 [5], which built on previous criteria such as the Revised American–European Consensus Group Criteria (AECG) [6] published in 2002. SjD criteria require evidence of specific autoimmunity in the form of anti-Ro (+/− anti-La) antibodies and/or histological evidence of focal lymphocytic sialadenitis along with clinical features of the condition. In most cases this would be objective evidence of reduced tear and/or saliva production, but in some cases this can be of extraglandular disease. Classification criteria are intended to define homogenous groups of patients suitable for research and so there will be some people who do not quite fit the classification criteria, perhaps because of a different autoantibody pattern or equivocal histological features where there is felt to be a high probability of the disease being present. Clinical practice is about uncertainty and probability (and flexibility), but the classification criteria generally underpin the approach to diagnosis and management guidelines in SjD [7]. Making an accurate diagnosis based on classification criteria may be a requirement for patient access to biological therapies, should they become available in the future.

## 3. Epidemiology

SjD is estimated to have a prevalence of circa 1 in 2500 adults [8]. Earlier studies had suggested a much higher prevalence, but this is likely to be due to looser criteria for the definition of the disease and different ascertainment methodology. It is about 16 times commoner in women than men for reasons that are not well studied, but are assumed to be due to an effect of female hormones on the immune system in the absence of an alternative explanation [9]. SjD typically starts in middle to older age groups (e.g., circa 40–60 years) and is thought to be of broadly similar prevalence in many different ethnic groups, although with some phenotypic variations [10].

## 4. Lip Biopsy and Histology

One obvious site for diagnostic biopsy in SjD would be the parotid gland, as the largest salivary gland. There are concerns, however, about the risk of damaging the facial nerve, causing facial paralysis, and so biopsy of the minor labial glands is more widely performed and generates comparable results [11]. It is generally a straightforward technique with a low frequency of side effects, particularly the linear incision approach in preference to punch biopsy, although there is still a modest risk of damage to local nerves with consequent long-term numbness/tingling/discomfort.

The interpretation and reporting of the biopsy is critical. The hallmark of SjD is of a periductal, focal, lymphocytic sialadenitis with a focus score of at least 1 [12]. Thus, it is both quantitative (a focus is a cluster/collection of at least 50 lymphocytes and a focus score of 1 means that there is at least one focus within a 4 mm^2^ section of tissue) and qualitative, i.e., there is a pattern to the location of foci in SjD that are located adjacent to salivary ducts (the location of SjD-related antigens driving the immune response?). This contrasts with normal glands with no obvious inflammation or glands with non-specific (non-SjD) inflammation, so-called ‘chronic sialadenitis’, in which a variety of inflammatory cells including plasma cells are scattered across the glands. Other parameters, such as the amount of tissue in the biopsy or how to interpret areas of atrophy or fibrosis, for example, may also be important, particularly in borderline cases [13].

In addition, it is helpful to know if there are any organized aggregates of B-cells forming ‘germinal-centre like structures’ along with T follicular helper cells and follicular dendritic cells, often identified by anti-CD21 immunohistochemical staining. If present, there is a higher likelihood of future mucosa-associated lymphoid structure (MALT) lymphoma development [14].

In writing or reading a biopsy report, therefore, it is important to assess whether the report includes reference to the pattern of inflammation and a focus score of 1 or more. If the report references ‘chronic inflammation’ or ‘chronic sialadenitis’, e.g., with diffuse plasma cell infiltration, with no reference to focal (periductal lymphocytic) inflammation, and concludes with a comment that ‘this may be compatible with a diagnosis of Sjogren’s Disease—correlate with clinical/serological features’, then this is a negative biopsy and this type of comment, although not inaccurate, risks confusion.

## 5. Symptom Burden, Psychological Impact, QoL, Health and Economic

In general, 90% of people with SjD report fatigue as a major feature and limb pain forms part of the symptom triad along with fatigue and dryness [15]. About 5% will have fibromyalgia in the UK [15], although much higher rates have been quoted in other populations [16,17,18]. Questionnaires do not necessarily identify a unique character to this fatigue, which has similarities to the chronic fatigue seen in SLE with both physical and mental fatigue (e.g., ‘brain fog’) increased compared with controls, whereas in rheumatoid arthritis, physical fatigue consequent to arthritis is more severe. Patients with SjD typically report day-to-day variability in fatigue severity, and so it may be better to assess it over a week or two-week period to gain an understanding of overall severity [19].

SjD patients also report reduced health-related quality of life (HRQoL), typically assessed by measures such as the EQ-5D or SF-36 questionnaires, and this has been a consistent finding across many studies [20]. Fatigue, pain and low mood/depression correlate more closely with HRQoL measures than either oral or systemic features, and this has implications in relation to justifying the cost of biologic therapies (see later chapters).

General HRQoL measures do not seem to capture the effect of severe dryness on affected individuals and so a SjD-specific HRQoL measure may be a better way of expressing this [21]. Some patients with severe eye and/or mouth dryness, particularly with high levels of dryness symptoms, can be badly affected with a major impact on their quality of life [22,23].

Having SjD also has financial impacts. Having the condition means more direct healthcare costs in the form of more visits to healthcare professionals, more investigations, and more dental and medication costs [24]. Although, typically, patients with SjD are in the later stages of their careers or are homeworkers, nevertheless, estimates of the indirect impact of SjD on work or caring activities have indicated that there are broader societal costs as well [25].

## 6. Systemic Disease (Including Oral and Ocular Elements)

Approximately 40% of SjD patients will have ‘objective’ features of systemic disease, i.e., considering this element separately to symptoms of fatigue or pain. The major systems involved are as follows and most of these can be captured through the EULAR Sjogren’s Syndrome Disease Activity Index (ESSDAI) [26] (see also later review). To put this another way, the development and validation of the ESSDAI was an effective way of identifying the key systems affected by SjD. The 12 domains of ESSDAI are as follows, with a section on additional system domains at the end. The frequencies set out in Table 1) are taken from published cohorts [27,28,29,30]. Treatment of these features will be addressed in a later review.

### 6.1. Constitutional

Some SjD patients report fevers and night sweats. These need to be distinguished from hormonal causes (patients will usually distinguish menopausal symptoms from SjD-related symptoms) or from infection or lymphoma.

It is important to investigate unexplained weight loss, as for any other individual, and not to assume that this is automatically due to SjD until causes such as bowel or other cancer have been excluded. Coeliac disease is likely to be more common in individuals with SjD and vice versa due to an overlapping genetic background, and so this diagnosis should also be considered [31].

### 6.2. Lymphadenopathy and Lymphoma

Reactive lymphadenopathy and splenomegaly can be found in a number of inflammatory rheumatic diseases, including SjD. If there are pathologic appearances on ultrasound scanning or other imaging and/or progressive enlargement, then haematological assessment and biopsy are indicated.

It has long been recognized that primary SjD is associated with mucosa-associated lymphoid tissue (MALT) B-cell lymphoma, typically in the salivary glands, with a lifetime risk of 5–10% [32]. This is usually associated with progressive firm salivary gland enlargement, which should prompt imaging and biopsy (N.B. an aspirate or needle biopsy may not be sufficient for this purpose). In most cases this is a relatively low-grade, often indolent lymphoma, and may be treated by observation, low-grade radiotherapy, or, in some patients, systemic chemotherapy such as R-CHOP (Rituximab, Cyclophosphamide, Hydroxydaunorubicin (doxorubicin), Oncovin (vincristine), and Prednisolone) or rituximab monotherapy [33]. It can recur in some patients. Risk factors for lymphoma development include anti-Ro antibody positivity, germinal centre-like structures on minor labial salivary gland biopsy at presentation, low complement C4 levels, salivary gland enlargement, neuropathy and cryoglobulinaemia [34]. Some patients develop a more aggressive form of lymphoma such as diffuse large B-cell lymphoma (DLBCL), which can be associated with increased mortality [35].

### 6.3. (Salivary) Glandular

Some patients have intermittent/variable/persistent parotid or other salivary gland swelling. This is considered part of the systemic inflammation associated with SjD and so is considered separately to glandular dryness (even though mechanistically this is also primarily believed to be due to inflammation).

Salivary duct blockage can be due to stricture, and sialography can be helpful to identify this. Acute infection of a salivary gland can be associated with pain, overlying redness, fever, lymphadenopathy and/or expression of pus from the salivary duct. The development of lymphoma is considered above. Other diseases associated with salivary gland swelling include sarcoidosis, IgG4-related disease, diabetic and alcoholic sialosis and diffuse infiltrative lymphocytosis syndrome due to HIV infection.

### 6.4. Articular

Joint and muscle pain is common in patients with SjD and the prevalence of fibromyalgia is also increased (see above). Some patients, however, develop a rheumatoid arthritis-like pattern of inflammatory arthritis. Treatment generally follows that of rheumatoid arthritis [36]. In some individuals (e.g., with anti-CCP antibodies) this may actually represent an overlap with rheumatoid arthritis.

### 6.5. Cutaneous

Typical skin problems in SjD include subacute cutaneous lupus erythematosus, usually associated with positive anti-Ro +/− anti-La antibodies and usually in sun-exposed areas. The other typical skin finding is of small vessel (leukocytoclastic) vasculitis, often in the form of palpable purpura +/− urticaria, also usually found in seropositive patients. Occasionally, this can be associated with cryoglobulinaemia, which, if not due to SjD, can be associated with hepatitis C virus exposure [37]. Target lesions (erythema multiforme) are also associated with SjD.

### 6.6. Pulmonary

SjD, like other inflammatory rheumatic disease, can be linked to interstitial lung disease (ILD) and this can be asymptomatic or relatively asymptomatic to start with. If patients have any symptoms such as shortness of breath or a cough, or if they have basal crackles on auscultation, then a chest X-ray and lung function tests +/− a high-resolution CT scan of the lungs need to be considered [38]. Given the advent of anti-fibrotic therapy, the benefits of early diagnosis are recognized more prominently. Although non-specific interstitial pneumonia (NSIP) is said to be the commonest subtype of ILD, other forms such as lymphocytic interstitial pneumonia (LIP), usual interstitial pneumonia (UIP) and organizing pneumonia (OP) can also occur [39].

Dryness of the bronchial tubes can cause a chronic cough in SjD patients, but again this can only be assumed once other causes such as ILD, infection or cancer have been excluded. Bronchiectasis is also more common in patients with SjD (see below).

### 6.7. Renal

Renal disease in SjD can be a diagnostic and therapeutic challenge [40]. Glomerulonephritis has been described in SjD and, if suspected, renal referral and a renal biopsy may be indicated. The more common renal abnormality is tubulointerstitial nephritis (TIN), typically in the form of (distal) renal tubular acidosis (RTA) [41]. If the serum bicarbonate is below 15 mEq/L and the urine pH is more than 6, this is a good indicator of the presence of RTA in a patient with SjD who requires sodium bicarbonate therapy [42]. A raised serum chloride is also typically found. Patients with RTA, high urinary calcium and low urinary citrate can develop nephrocalcinosis and kidney stones. Hypokalaemia also needs to be watched out for. Progression of renal disease to end-stage renal disease and dialysis is relatively uncommon, although it can occur [43].

### 6.8. Muscular

Autoimmune myositis is rare in SjD (see Table 1). If it occurs, it should be diagnosed and managed according to standard approaches [44]. A subset of patients with inclusion body myositis associated with anti-Ro antibodies (especially anti-Ro52) has been recognized [45] and anti-Ro52 antibodies have also been linked to interstitial lung disease in patients with idiopathic myositis [46].

### 6.9. Peripheral Nervous System

Small-fibre sensory neuropathy with normal ‘standard’ nerve conduction studies is common in SjD [47]. Small-fibre neuropathy can be further investigated with specialist tests such as a cutaneous biopsy, and/or altered or absent laser-evoked potentials and/or abnormal quantitative sensory testing to thermal stimuli and/or abnormal sympathetic sensory testing. In clinical practice it may be assumed through the combination of symptoms such as pain, tingling and/or numbness and exclusion of large fibre neuropathy through the presence of normal conventional nerve conduction studies.

Large-fibre distal sensory polyneuropathy, axonal sensorimotor polyneuropathy, chronic inflammatory demyelinating polyneuropathy (CIDP), multiple mononeuritis, trigeminal neuropathy, ganglionopathy and cryoglobulin-related vasculitic neuropathy have all been described in SjD [48]. Sensorimotor polyradiculoneuropathy corresponding to Guillain–Barré syndrome has also been described.

### 6.10. Central Nervous System (CNS)

An increase in non-specific white matter lesions in the brain on MRI scanning is common in SjD and is of uncertain clinical significance [49]. Otherwise, CNS involvement in SjD is rare but potentially serious when it does occur. Conditions include transverse myelitis, optic neuritis, other central cranial nerve involvement, multiple sclerosis (MS)-like disease, seizures, aquaporin-4 antibody-related (Devic’s) disease, lymphocytic meningitis and cerebral vasculitis. In the case of optic neuritis and MS, it may be somewhat arbitrary as to whether these are regarded as associated or independent conditions [50]. Cognitive disorder (‘brain fog’) is generally considered to be part of a fibromyalgia complex, which may be linked to other neurological conditions, SjD, or both, rather than being a feature of CNS per se, unless there is a clear temporal association with CNS features.

### 6.11. Haematological

Autoimmune cytopenias, particularly neutropenia, are common in SjD [51]. Lymphopenia and Idiopathic thrombocytopenic purpura (ITP) are also associated with SjD, as is autoimmune haemolytic anaemia.

### 6.12. Biological

A polyclonal raised IgG level (+/− raised IgA or IgM) is common in SjD (typically above 16 g/L or above 20 g/L). Monoclonal gammopathy of undetermined significance (MGUS) is also increased [52] and can be linked with salivary gland swelling, vasculitis, neurological features, rheumatoid factor positivity, low complements, cryoglobulinaemia and a greater risk of lymphoma development [53].

### 6.13. Other Systemic Features Not Captured Through the ESSDAI

A large international cohort study [54] identified that over a quarter of patients with primary SjD may have systemic manifestations not currently included in the ESSDAI classification, although other than Raynaud’s phenomenon, found in a third of patients with SjD [55], the individual frequency of each of these non-ESSDAI features was very low. These include:

Gastrointestinal involvement, which ranges from presumed dryness-related irritable bowel syndrome and motility type issues to varying types of local surface tissue/systemic inflammation (dysphagia, chronic gastritis, protein-losing enteropathy or gastrointestinal tract vasculitis) [56].

Sjögren’s can also be associated with liver conditions such as primary biliary cholangitis, autoimmune hepatitis and mild elevation of liver enzymes [57]. Pancreatic involvement can include subclinical issues, but pancreatitis has also been reported, albeit rarely. Pernicious anaemia has also been reported.

Autonomic nervous system involvement is well reported and can be associated with blood pressure and heart rate regulation causing postural hypotension, dysregulated sweating, gastrointestinal symptoms or bladder problems [58].

Cardiovascular disease has also been described (heart valve disease, myocarditis, pericarditis) as well as an increased risk of cardiovascular and cerebrovascular disease, presumed to be due to the increase in systemic inflammation in some patients [59]. Whether anti-Ro antibodies are associated with electrocardiological abnormalities is unclear [60,61].

Other pulmonary involvement includes bronchiectasis, presumed to be due to underlying inflammation of the tissue leading to damage [62]. Pleurisy/pleural effusions and pulmonary artery hypertension have also been described.

Ear, nose and throat involvement includes an association with acute hearing loss [63], laryngitis, sinusitis and relapsing polychondritis.

Other cutaneous associations include erythema nodosum, granuloma annulare [64], panniculitis and amyloidosis [65].

Urological involvement, specifically interstitial cystitis, is well recognized [66].

Other eye conditions apart from dryness include uveitis, scleritis and episcleritis [67].

## 7. Principles of Investigation and Treatment

Treatment of SjD will be addressed in detail in later chapters. Some key general principles include: (1) to make a diagnosis of SjD using the AECC wherever possible (e.g., see British Society for Rheumatology Guidelines, described in ‘Diagnosis and Classification’ section above); (2) not to assume in patients with SjD that their symptoms are automatically due to SjD. Patients with SjD can develop breast or bowel cancer or other disease just as anyone else can, and should be investigated accordingly if they present with new symptoms; (3) to optimize simple interventions such as oral and eye health; (4) to involve patients in management decisions; and (5) to consider a checklist including general areas that can be overlooked such as psychological support, pregnancy and sexual health, and cancer, osteoporosis and cardiovascular risk assessments.

Having considered these principles, therapeutic interventions can then follow guidelines such as those produced by the British Society for Rheumatology [7] or by EULAR [68] or others. Pilocarpine can be considered in individuals with moderate/severe dryness but who still have residual (stimulable) glandular function, and hydroxychloroquine is typically offered to patients with arthralgia/pain/fatigue and/or mild/moderate systemic features. The basis for using stronger immunosuppression will be dealt with in later reviews.

## 8. Disease Heterogeneity and Stratification

SjD is a heterogenous condition, ranging from individuals with predominantly dryness features to individuals with fatigue/pain predominant and/or patients with varying combinations of systemic disease including lymphoma. Stratifying patients has been an area of active interest, e.g., by symptoms [69] or biological process [70], or by multiple clinical and investigational parameters [71]. From a clinical perspective, the divisions into seronegative/seropositive, systemic features/non-systemic features, low lymphoma risk/higher lymphoma risk, and low symptom burden/high symptom burden are probably the most useful considerations within these various schemes.

## 9. Prognosis and Outcome

Most patients living with SjD will have a normal lifespan [72]. The exceptions will be a small percentage of individuals who develop more severe forms of lymphoma or other systemic disease requiring strong immunosuppression [73]. There may also be a small increased risk of death related to cardiovascular disease (see above). Overall, however, the standardized mortality rate in SjD is increased compared with the general population [74].

## 10. Vaccination, Pregnancy and Surgery

There is a general consensus that patients with SjD should receive ‘standard’ vaccinations such as the annual flu and pneumococcal vaccinations and others as indicated, such as having a tetanus booster. When it comes to ‘live’ vaccines, this depends on whether the individual has mild SjD and is considered ‘immunocompetent’ or whether they are on immunosuppressant medication such as higher doses of prednisolone, methotrexate or mycophenolate, rituximab or others, or have another reason to be considered ‘immunocompromised’, in which case they may be advised against having such vaccines. In this situation it is best to seek medical advice and err on the side of caution. The Sjogren’s Foundation has advice directed at patients in the United States of America on their website and the NHS also has general advice on vaccination.

There are limited data on whether women with SjD have difficulty in becoming pregnant (reduced fertility) [75]. They may, however, be more likely to have pregnancy complications and there may be some increase in pregnancy loss [76]. One critical issue is to advise women of childbearing age with anti-Ro/La antibodies of the potential for and need to monitor the foetal heart rhythm from week 16 to detect congenital heart block [77]. Specialist advice and guidance is necessary should this develop. Hydroxychloroquine is recommended for secondary prevention [78] and there is a move to use it for primary prevention in women known to have anti-Ro/La antibodies, although the evidence for this is not as clear-cut [79].

In terms of surgery in patients with SjD, the main principle is of awareness and taking additional care. Generally, patients with SjD who are not immunocompromised do not appear to be at greater risk of postoperative complications or hospital mortality [80]. If they are having cataract surgery, then careful attention to surface lubrication before and after surgery is advised [81]. If they are immunosuppressed, however, then appropriate local/national protocols should be followed and, if they have autonomic neuropathy or pulmonary or cardiovascular disease, the appropriate mitigations should be put in place, as for any other person with these conditions.

## 11. Patient Perspectives

The top priorities for patients in a study in the United States of America in terms of treatment were to improve symptoms of fatigue, dryness and pain, address the social burden of living with SjD and reduce lymphoma risk [82]. Sex life, social activities and work were the main areas important for quality of life. Healthcare costs, particularly dental care costs, were a financial concern. Similarly, Vieira et al. 2021 [83], on behalf of Sjögren Europe, emphasized that fatigue and cognitive dysfunction cause the greatest patient-reported disability and are a major unmet need. Patients also report that they do not always feel that the medical profession recognizes the seriousness or impact of SjD on their lives [84].

## 12. Conclusions

Although it is important to optimize the management of oral, ocular and other dryness features in SjD, it is also important to recognize that SjD can be a serious, multi-system autoimmune disease that can be debilitating and even life-threatening for some patients. This understanding of the many different presentations and systemic complications of SjD, as set out in this chapter, supports accurate and timely diagnosis, in turn hopefully leading to improved clinical management and patient outcomes, as discussed in the following reviews.

## Figures and Tables

**Table 1:** Prevalence of systemic disease by ESSDAI domain in four cohorts of patients with SjD. N.B. Presented as a range of values for those registries with subgroup data published.

Organ System	UKPSSR (UK)	ASSESS (France)	Big Data (International)	GEAS-SS (Spain) (Any Time Point)
	*n* = 665	*n* = 395	*n* = 10,500	*n* = 921
Constitutional	22.5%	4.1%	9.3–11.1%	8.5–11.7%
Lymphadenopathy and Lymphoma	5.4%	2.4%	9.2–10.3%	6.5–10.2%
Salivary Glandular	18.5%	12.1%	22.4–24.5%	28–33.9%
Articular	31.7%	18.6%	37.1–39.7%	40.2–55.9%
Cutaneous	8.0%	4.2%	11.0–12.5%	8.6–13.4%
Pulmonary	8.7%	14.4%	10.5–11.4%	6.1–15.0%
Renal	2.9%	2.8%	5.0–6.3%	1.8–4.3%
Muscular	1.7%	3.3%	2.3–3.0%	0.5–1.4%
Peripheral Nervous System	4.4%	9.6%	6.0–6.2%	5.1–10.4%
Central Nervous System	0.3%	2.0%	1.8–2.0%	1.8–2.7%
Haematological	16.3%	15.6%	25.5–28.3%	Not provided in this format
Biological	47.4%	37.4%	56.9–64.6%	Not provided in this format

UKPSSR: United Kingdom Primary Sjogren’s Syndrome Registry; ASSESS: Assessment of Systemic Signs and Evolution of Sjogren’s Syndrome; GEA-SS: Spanish Group for the Study of Sjögren’s Syndrome.

## Data Availability

Not applicable.
